# Risk of Cesarean Delivery after Vaginal Inserts with Prostaglandin Analogs and Single-Balloon Catheter Used for Cervical Ripening and Induction of Labor

**DOI:** 10.3390/biomedicines11082125

**Published:** 2023-07-27

**Authors:** Maciej W. Socha, Wojciech Flis, Miłosz Pietrus, Mateusz Wartęga

**Affiliations:** 1 Department of Perinatology, Gynecology and Gynecologic Oncology, Faculty of Health Sciences, Collegium Medicum in Bydgoszcz, Nicolaus Copernicus University, 85-821 Bydgoszcz, Poland; 2 Department of Obstetrics and Gynecology, St. Adalbert’s Hospital in Gdańsk, Copernicus Healthcare Entity, 80-462 Gdańsk, Poland; 3 Department of Gynecology and Oncology, Jagiellonian University Medical College, 31-501 Kraków, Poland; 4 Department of Pathophysiology, Faculty of Pharmacy, Collegium Medicum in Bydgoszcz, Nicolaus Copernicus University, 85-094 Bydgoszcz, Poland

**Keywords:** cesarean section, delivery, obstetric, dinoprostone, labor, induced, misoprostol

## Abstract

(1) Background: Induction of labor is currently the most frequently performed procedure in modern obstetrics, referring to more than one in five women, and it is postulated that the percentage of labor induction will increase. (2) Methods: In total, 2935 patients in uncomplicated full-term pregnancy fulfilled the study inclusion criteria and underwent induction of labor. Pregnant women were divided into three groups: IOL with Dinoprostone, Misoprostol vaginal suppositories, and Foley catheter. Outcomes, including cesarean section rates, time to delivery, and cesarean section indications, were analyzed. (3) Results: There was statistically significantly more cesarean sections within 24 h in the Misoprostol group in comparison with the Dinoprostone and Foley catheter groups (*p* < 0.0001). The percentage of patients who had a cesarean section due to clinically diagnosed threatened fetal asphyxia was 63% in the Dinoprostone group, 81.3% in the Misoprostol group, and 55.3% in the Foley catheter group. There were statistically significantly more cesarean deliveries due to nonreassuring fetal heart rate patterns within 24 h in the Misoprostol group in comparison with the Dinoprostone and Foley catheter groups (*p* = 0.0031 and *p* = 0.0363). (4) Conclusions: Misoprostol may cause a more turbulent and violent course of labor, with a higher rate of increased incidence of nonreassuring fetal heart rate patterns and cesarean deliveries. The use of a Dinoprostone vaginal insert or Foley catheter may provide longer labor, although still within 48 h, with a lower risk of cesarean section caused by nonreassuring fetal heart rate patterns.

## 1. Introduction

One of the commonly used procedures in modern obstetrics is the induction of labor (IOL). This procedure is defined as the iatrogenic triggering of uterine contractions, leading to vaginal delivery within 24–48 h. Over 20% of pregnant women undergo induction of labor procedures due to fetal and maternal indications [1,2]. IOL protocol mainly consists of cervical preparation with a cervical ripening agent (due to unfavorable cervix) followed by intravenous oxytocin infusion. It is postulated that the percentage of labor induction will increase in the world of obstetrics [3]. In current global obstetrics, there are many methods of induction of labor, which aim to both prepare the cervix and trigger contractions. In the case of a prepared, ripened cervix, amniotomy with simultaneous intravenous oxytocin infusion is a popular method of labor induction. However, in the case of an unprepared cervix, other methods are used, such as pharmacological agents or mechanical devices. Pharmacological methods include prostaglandin analogs (mainly prostaglandin E1 and E2 analogs) and progesterone antagonists [2]. In turn, mechanical methods include the Foley catheter or Cook’s double-balloon catheter (DBC). From the point of view of successful labor induction, it is crucial to perform IOL quickly and effectively. An additional challenge is the successful induction of labor in women with an unprepared cervix. The ideal agent for the induction of labor should be primarily effective, safe, and lead to successful vaginal delivery in most cases within the time frame that meets the definition of labor induction.

Most commonly used agents for cervical ripening are prostaglandin analogs (PGs) or mechanical devices—single-balloon catheters (Foley catheters) [4,5]. The most commonly used prostaglandin analogs are Dinoprostone (prostaglandin E2 analog) and Misoprostol (prostaglandin E1 analog) [6]. Both PGs analogs have a similar mechanism of action with different pharmacokinetic profiles depending on the route of administration [7]. The Foley catheter, being a very cheap device, participates in cervical ripening mainly by mechanical dilation of the cervical canal. In addition, the Foley catheter may enhance the release of endogenous prostaglandins due to the mechanical stretching of the fetal membranes and cervical tissue [6,8]. Each of these IOL methods has a different effectiveness and safety profile. 

Pregnancy (especially the perinatal period) is a period of increased stress for patients. In addition to effective and safe action, the agent used to induce labor should also be associated with patient satisfaction with the induction method used. Cesarean section (CS) is the oldest known obstetrical surgery. The latest available data show that approximately 21.1% of women have given birth by cesarean worldwide, with a steady increase worldwide which will continue increasing over the current decade [9,10,11]. As the CS rate has increased, maternal mortality and morbidity have also risen steadily over the last few years [10]. Our clinical observations show that patients’ awareness of possible complications has significantly increased in recent years. Therefore, the most common questions asked by patients who were offered selected methods of induction of labor concern the risk and reasons for cesarean delivery. 

Currently, there is an ongoing discussion about the best methods of labor induction. Despite many studies being conducted in this area, it has not been possible to clearly determine which of the methods is the most effective and safest. That is why we decided to conduct a retrospective observational study to clearly determine how the use of most commonly used methods of IOL translates into cesarean section rates. 

The aim of this study is to evaluate the CS rates after the application of vaginal inserts containing prostaglandin E1 and E2 analogs (Misoprostol and Dinoprostone) and a single-balloon catheter (Foley catheter) as the most commonly used methods of labor induction, with the emphasis on the CS indications. This single-center retrospective observational study will translate the statics into an answer to the clinical concerns of a particular patient.

## 2. Materials and Methods

This is an observational, retrospective single-center study. The study was conducted in accordance with the Declaration of Helsinki and approved by the Institutional Ethics Committee of Collegium Medicum in Bydgoszcz, Nicolaus Copernicus University. The medical records of 11736 pregnant women who had delivered at the Department of Obstetrics and Gynecology, St. Adalbert’s Hospital in Gdańsk, Poland (a tertiary referral hospital) between 1 June 2019 and 31 December 2022 were analyzed. Data on the course of labor were recorded in a computer database during the patient’s stay. During the analysis of the previously obtained documentation, the following information was achieved: duration of pregnancy (determined on the basis of the date of the last menstruation and confirmed by the first-trimester ultrasonography), type of cervical ripening agent used for induction of labor, course of delivery, duration of labor, parturition type, previous obstetric history, and patient demographic data. The previously acquired dataset was checked for inconsistencies and any possible errors.

The criteria for inclusion in the study group were effective labor induction, use of Misoprostol or Dinoprostone vaginal inserts or Foley catheter as labor induction methods, uncomplicated full-term pregnancy (>41 weeks of gestation), single live pregnancy, no previous cesarean sections, and cephalic fetal presentation.

The exclusion criteria were spontaneous labor, previous cesarean section, premature rupture of membranes (PROMs), pre-term delivery, twin pregnancy, complications of pregnancy necessitating earlier induction of labor, lack of critical information in medical documentation, and any contraindications to vaginal delivery and induction of labor.

The study included patients qualified for induction of labor for both maternal and fetal indications and with an unfavorable cervix (Bishop score < 6 and >2). Indications for IOL were in accordance with the current recommendations of the Polish Society of Gynecologists and Obstetricians [12].

**Figure 1:**
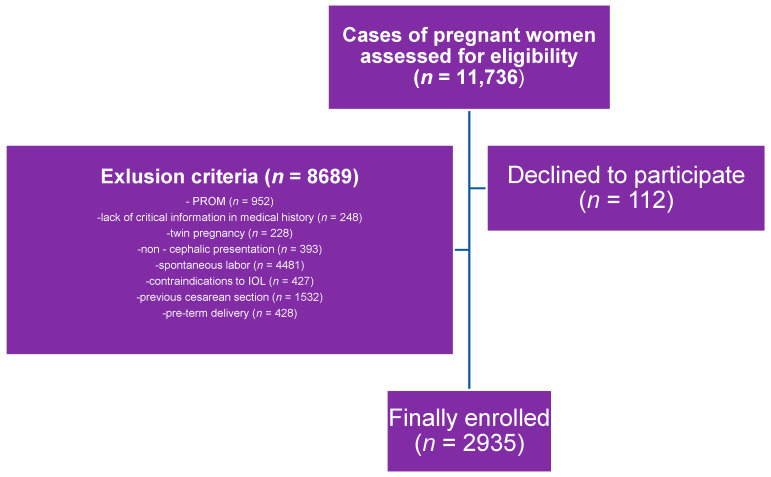
Inclusion and exclusion criteria for the study group.

An analysis of the documentation covering 11,736 deliveries at the analyzed time was carried out, of which, based on the above-described criteria, 2935 cases were qualified for analysis (Figure 1). All the patients were adults.

Depending on the agent used, the induction of labor took the following protocol: 200 micrograms of Misoprostol or 10 milligrams of Dinoprostone in the form of vaginal inserts or single-balloon catheter in the form of Foley catheter insertion above the internal ostium of the cervical canal. Once the Foley catheter had passed through the cervical canal, the catheter balloon was filled with sterile saline to a volume of 80–100 mL.

We assessed the effectiveness in labor induction of popularly used IOL agents. The primary outcome was the rate of cesarean section depending on the agent used to induce labor. The secondary outcome was the analysis of the indications for cesarean delivery, such as lack of progress in labor (LoPiL) and nonreassuring fetal heart rate patterns (NRFHRP), suggesting threatened fetal asphyxia. The tertiary endpoint was the time for cesarean delivery.

### Statistical Methodology

Statistical analyses were performed using the statistical suite Statistica software version 14 (StatSoft. Inc., Hamburg, Germany) and Microsoft Excel 2013 (Microsoft, Redmond, WA, USA).

The quantitative variables were characterized by the arithmetic mean of standard deviation or median or max/min (range) and a 95% confidence interval. The qualitative variables were presented with the use of count and percentage.

In order to check if a quantitative variable derives from a population of normal distribution, the W Shapiro–Wilk test was used, whereas to prove the hypotheses on the homogeneity of variances, the Leven (Brown–Forsythe) test was utilized.

The statistical significance of differences between the two groups was processed with Student’s *t*-test or Mann–Whitney U-test. The significance of the difference between more than two groups was assessed using the F test (ANOVA) or Kruskal–Wallis test. In the case of statistically significant differences between the two groups, post hoc tests were utilized (Tukey’s test for F or Dunn for Kruskal–Wallis).

In the case of two paired variables, a model Student’s *t*-test or Wilcoxon signed-rank test was utilized. The significance of the difference between more than two variables in the paired variables model was checked using an analysis of variance with repeated measurements or the Friedman test.

Chi-squared tests for independence were used for qualitative variables. In order to determine dependence, strength, and direction between variables, correlation analysis was performed by determining the Pearson or Spearman’s correlation coefficients.

In all the calculations, a statistical significance level of *p* = 0.05 was used.

## 3. Results

**Figure 2:**
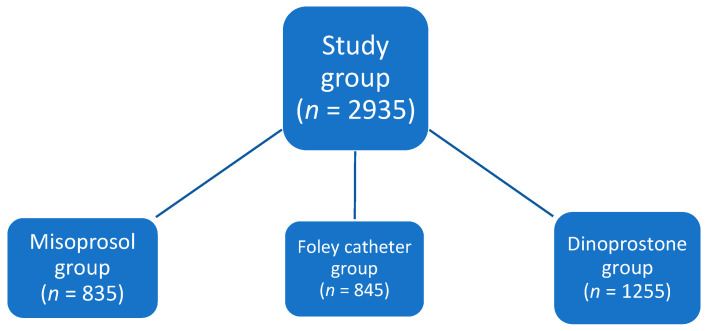
Division of patients into three groups depending on the agent used for the IOL.

A total of 2935 patients (in uncomplicated full-term pregnancy) were finally enrolled in this study group. Subsequently, pregnant patients were divided into three groups depending on the IOL agent administered: Dinoprostone vaginal insert group (*n* = 1255), Misoprostol vaginal insert group (*n* = 835), and Foley catheter group (*n* = 845) (Figure 2). The age of the patients ranged from 21 to 38 years, with the median age of the participants being 29 years. In total, 1819 patients (63%) were primiparas, and the remaining 1116 (37%) were multiparous patients.

Out of a total of 2935 patients, 87.6% (*n* = 2571) delivered vaginally. The CS rates for each group were 11% (*n* = 138) for the Dinoprostone group, 18% (*n* = 150) for the Misoprostol group, and 9% (*n* = 76) for the Foley catheter group. Respectively, the rates of vaginal deliveries were 89% (*n* = 1117), 82% (*n* = 685), and 769 (*n* = 91%) (Table 1).

There were statistically significantly more cesarean deliveries in the Misoprostol group than in the Dinoprostone group (*p* < 0.0001). Additionally, there were significantly more CS in the Misoprostol group than in the Foley catheter group (*p* < 0.0001).

No statistically significant differences were found between the Dinoprostone and Foley catheter groups (*p* = 0.1370).

Considering CS, most of the examined patients delivered within 48 h (51.9%, *n* = 189).

The percentages of women who underwent cesarean delivery within 24 h in the Dinoprostone, Misoprostol, and Foley catheter groups were 26.1% (*n* = 36), 71.3% (*n* = 107), and 42.1% (*n* = 32) (Table 2).

Respectively, considering CS within 48 h, the percentages were 73.9% (*n* = 102), 28.7 (*n* = 43), and 57.9% (*n* = 44).

There were statistically significantly more cesarean sections within 24 h in the Misoprostol group in comparison to the Dinoprostone and Foley catheter groups (*p* < 0.0001). Additionally, there were statistically significantly more cesarean deliveries within 24 h in the Foley catheters group than in the Dinoprostone group (*p* = 0.0160).

The percentages of patients who underwent a CS due to nonreassuring fetal heart rate patterns were 63% (*n* = 87) in the Dinoprostone group, 81.3% (*n* = 122) in the Misoprostol group, and 55.3% (*n* = 42) in the Foley catheter group. For each group, the rate of lack of progression in labor (as an indication for cesarean delivery) was 37% (*n* = 51), 18.7% (*n* = 28), and 44.7% (*n* = 34), respectively (Table 3).

There were statistically significantly more cesarean deliveries due to nonreassuring fetal heart rate patterns suggesting threatened fetal asphyxia in the Misoprostol group in comparison with the Dinoprostone group (*p* = 0.0005) and the Foley catheter group (*p* < 0.0001).

No statistically significant differences were found between the Dinoprostone and Foley catheter groups for those variables (*p* = 0.2657).

The percentages of women who underwent cesarean delivery due to nonreassuring fetal heart rate patterns within 24 h in the Dinoprostone, Misoprostol, and Foley groups were 72.2% (*n* = 26) vs. 91.6% (*n* = 98) vs. 78.1% (*n* = 25) compared to women who underwent cesarean section due to lack of progression in labor within 24 h, for which the percentages were 27.8% (*n* = 10) vs. 8.4% (*n* = 9) vs. 21.9% (*n* = 7), respectively (Table 4).

There were statistically significantly more cesarean deliveries due to nonreassuring fetal heart rate patterns within 24 h in the Misoprostol group in comparison with the Dinoprostone and Foley catheter groups (*p* = 0.0031 and *p* = 0.0363).

No statistically significant differences were found between the Dinoprostone and Foley catheter groups (*p* = 0.5747).

The percentages of women who underwent cesarean delivery due to nonreassuring fetal heart rate patterns within 48 h in the Dinoprostone, Misoprostol, and Foley groups were 59.8% (*n* = 61) vs. 55.8% (*n* = 24) vs. 38.6% (*n* = 17) compared to women who underwent cesarean section due to lack of progression in labor within 48 h, for which the percentages were 40.2% (*n* = 41) vs. 44.2% (*n* = 19) vs. 61.7% (*n* = 27), respectively (Table 5).

There were statistically significantly more cesarean deliveries due to nonreassuring fetal heart rate patterns in the Dinoprostone group in comparison with the Foley catheter group (*p* = 0.0186).

No statistically significant differences were found between Dinoprostone and Misoprostol groups (*p* = 0.6559) and between the Foley catheter and Misoprostol groups (*p* = 0.6559).

## 4. Discussion

The hunt for a safe and efficacious induction of labor has received widespread attention and is a subject of ongoing thorough discussion. Cesarean section as an emergency procedure is a life-saving intervention for the mother or fetus. However, it can also lead to short- and long-term consequences for both the woman and her newborn baby. Maternal complications include a longer recovery time after operational delivery, compared to vaginal, which translates into an increased percentage of postpartum depressive symptoms. In addition, somatic complications affecting subsequent pregnancies may cause uterine rupture, scar pregnancy, or abnormal placentation to occur [13,14].

It is also worth mentioning the complications of the newborn due to cesarean delivery—transient tachypnea of the newborn, surfactant deficiency, and pulmonary hypertension. Long-term complications include altered immune development and the increased likelihood of allergy, atopy, asthma, and obesity [15].

Currently, about one in five women undergo labor induction worldwide, with an increasing trend. About 20–21% of deliveries end by cesarean section. An increase in the percentage of labor induction (for both maternal and fetal indications) is predicted, which will undoubtedly also affect the percentage of cesarean sections [2,16].

Most publications agree that labor induction is associated with an increased risk of cesarean delivery [17,18,19]. Currently, global obstetrics is aiming to reduce the percentage of cesarean sections. Considering the number of labor inductions worldwide, it is worthwhile to clearly establish the link between the agents used in labor induction and answer how this translates to cesarean section risk. The aim of this study was to evaluate the rate of cesarean sections after the application of commonly used agents (Misoprostol or Dinoprostone vaginal inserts and Foley catheter) in labor induction, with an emphasis on the cesarean section indications and time to cesarean delivery. This date aims to answer the questions of patients to whom IOL is offered, regarding risk and reasons for cesarean delivery with labor induction.

Our study undoubtedly proves the effectiveness of prostaglandin analogs and Foley catheters in labor induction, understood as effective vaginal delivery within 24–48 h. The vast majority of patients in each group delivered vaginally, and a small percentage of patients delivered by CS (11%, 18% and 9%). However, this study focuses on cesarean deliveries—vaginal births are being reviewed by other studies conducted by our team. These data are consistent with previous studies evaluating the effectiveness of the above agents in labor induction. The ideal protocol for induction of labor should have a significant effect on cervical ripening, trigger uterine contractions, and have the lowest CS rate. Additionally, it should have as few side effects as possible [20].

Interestingly, the dominant group in terms of the percentage of cesarean sections was the group of patients who were induced with Misoprostol (18%). In addition, we showed statistical significance for a higher rate of cesarean deliveries in the Misoprostol vaginal insert group compared to the Dinoprostone Vaginal insert group and the Foley catheter group (*p* < 0.0001). The presented data are consistent with previous research in this field [21,22,23,24]. On the other hand, the lowest percentage of cesarean sections occurred in the Foley catheter group (9%).

Considering the time to cesarean delivery, the majority of patients who received the Misoprostol insert delivered within 24 h (71.3%). We also showed statistical significance for the increased percentage of cesarean sections within 24 h in the group of patients who receive Misoprostol compared to the other groups (*p* < 0.0001).

From the above data, it is clear that the use of the insert with Misoprostol is characterized by a higher percentage of cesarean sections and reduced time to cesarean delivery (most patients deliver within 24 h). These data are consistent with the results of the research carried out so far [21,25,26]. Most patients who received a Dinoprostone insert or Foley catheter delivered within 48 h with Dinoprostone dominance (73.9% and 57.9%). However, when considering delivery within 24 h, slightly more patients delivered in the Foley catheter group than in the Dinoprostone group, which was statistically significant (42.1% vs. 26.1%, *p* = 0.0160).

The dominant indication for CS in each of the study groups was nonreassuring fetal heart rate patterns (63% vs. 81.3% vs. 55.3%). It is worth noting that although nonreassuring fetal heart rate patterns are the dominant indication for cesarean section in all study groups, the percentage of indications varies significantly depending on the time to delivery. Within 24 h, the main indication for cesarean section was a nonreassuring fetal heart rate pattern, clinically interpreted as threatened fetal asphyxia (72.2%, 91.6%, and 78.1%), with a very low rate of and lack of progress in labor. On the other hand, in the period up to 48 h, despite the slight dominance of nonreassuring fetal heart rate patterns, the percentage of lack of progression in labor as an indication for cesarean section increases (40.2%, 44.2%, and 61.4%). We believe that the higher rate of cardiotocographic changes diagnosed as threatening fetal asphyxia within 24 h may also be due to tachysystole [27,28]. Uterine hyperstimulation can often be confused with an abnormal CTG record (nonreassuring fetal heart rate patterns), which was the main indication for cesarean section. In the case of abnormal cardiotocography (nonreassuring fetal heart rate) that accompanies abnormal contractile function, the diagnosis of threatening fetal asphyxia, not hyperstimulation, is most important for the clinician. Therefore, apart from the lack of progress in labor, the dominant indication for cesarean section was nonreassuring fetal heart rate patterns. From a practical point of view, it is worth assuming that in this group of patients, some cases may have been accompanied by hyperstimulation, but it is impossible to confirm this at the present stage of the analysis. Therefore, we believe that it is worth conducting additional studies that, in addition to the rate of cesarean sections, will also assess the amount of hyperstimulation after the use of these prostaglandin analogs. In addition, lack of labor progression as an indication for a cesarean section does not predominate within 48 h. We believe that phenomenon can be explained by the significant effect of the IOL agents on the cervical tissue.

Prostaglandin analogs are directly involved in cervical ripening by mediating biochemical pathways in the cervix. The Foley catheter, in addition to mechanical dilation of the cervical canal, has the ability to stimulate the secretion of endogenous prostaglandins from the cervical tissue and fetal membranes [5,6].

We believe that due to their properties, presented IOL agents significantly prepare the cervix for delivery, which translates into a more harmonious delivery, reducing the risk of cervical dystocia, which translates into a lower rate of lack of progression in labor.

In conclusion, a clear pattern emerges from the above data. The use of a Misoprostol vaginal insert is characterized by the highest percentage of cesarean sections among the tested agents. In addition, after Misoprostol administration, the highest percentage of patients delivered within 24 h, where nonreassuring fetal heart rate patterns indicating a clinical diagnosis of threatening fetal asphyxia was the dominant indication for cesarean section. We believe that the use of Misoprostol, despite the shorter time to cesarean delivery, may result in a more violent and traumatic course of childbirth.

Patients who receive Dinoprostone vaginal insert or a Foley catheter have a lower cesarean section rate. The dominant indication for cesarean section in these groups was still threatening fetal asphyxia (in the form of nonreassuring fetal heart rate patterns) but with a lower rate than within 24 h.

Based on the above data, we believe that the use of Dinoprostone and the Foley catheter in patients with full-term pregnancy is characterized by a much more peaceful and harmonious delivery, which may translate into the patient’s wellbeing and satisfaction.

Our study is retrospective and purely clinical. We assessed the patients from a clinical point of view, and on this basis we draw conclusions. Undoubtedly, our study has its limitations. Our study aimed to retrospectively assess the rates of cesarean deliveries depending on the most commonly used IOL agents (Foley catheter and prostaglandin analogs). A definite limitation of our study is the lack of a thorough analysis of the results depending on the parity of the patients. Moreover, due to the retrospective nature of our study, the results should be interpreted cautiously as there is no control group. Additionally, we believe that an important limitation of our study may be the fact that the analysis included only patients in full-term patients (>37 weeks of gestation) with uncomplicated pregnancies without taking into account patients with pre-term delivery. Finally, no postnatal evaluation of the newborn’s condition was performed, taking into account possible complications. However, we believe that due to some limitations of our study, the results may contribute to the ongoing discussion regarding the best IOL methods. We also strongly believe that the results we present may prove to be an excellent starting point for further research in this field which should take into account numerous additional variables.

## 5. Conclusions

Our retrospective study enhances our understanding of using popular IOL agents and translates them into obstetrical practice. Our results provide positive evidence for the use of both prostaglandin E1 and E2 analogs and single-balloon catheter, showing that only a small percentage of deliveries end in cesarean section. Additionally, the use of a Dinoprostone vaginal insert or Foley catheter may provide longer labor, although delivery still occurs within 48 h, as indicated in the definition. At the same time, this use is associated with a lower risk of cesarean section caused by nonreassuring fetal heart rate patterns.

## Figures and Tables

**Table 1:** Comparative analysis in three groups (Dinoprostone, Misoprostol, and Foley catheter): parturition type.

	Dinoprostone (*n* = 1255)	Misoprostol (*n* = 835)	Foley (*n* = 845)	All (*n* = 2935)	*p*-Value
Parturition type:					<0.0001 ^1^
Vaginal delivery (VD)	1117 (89.0%)	685 (82.0%)	769 (91.0%)	2571 (87.6%)	
Cesarean section (CS)	138 (11.0%)	150 (18.0%)	76 (9.0%)	364 (12.4%)	

^1^ Chi-square.

**Table 2:** Comparative analysis in three groups (Dinoprostone, Misoprostol, and Foley catheter): time to cesarean section delivery.

	Dinoprostone (*n* = 138)	Misoprostol (*n* = 150)	Foley (*n* = 76)	All (*n* = 364)	*p*-Value
					<0.0001 ^1^
Up to 24 h	36 (26.1%)	107 (71.3%)	32 (42.1%)	175 (48.1%)	0.0160 ^1^
Up to 48 h	102 (73.9%)	43 (28.7%)	44 (57.9%)	189 (51.9%)	

^1^ Chi-square.

**Table 3:** Comparative analysis in three groups (Dinoprostone, Misoprostol, and Foley catheter): cesarean section indications (nonreassuring fetal heart rate patterns (NRFHRP) and lack of progress in labor (LoPiL).

CS Indication	Dinoprostone (*n* = 138)	Misoprostol (*n* = 150)	Foley (*n* = 76)	All (*n* = 364)	*p*-Value
					0.0005 ^1^
NRFHRP	87 (63.0%)	b122 (81.3%)	42 (55.3%)	251 (69.0%)	<0.0001 ^1^
LoPiL	51 (37.0%)	28 (18.7%)	34 (44.7%)	113 (31.0%)	

^1^ Chi-square.

**Table 4:** Comparative analysis in three groups (Dinoprostone, Misoprostol, and Foley catheter: cesarean section indications within 24 h (nonreassuring fetal heart rate patterns (NRFHRP) and lack of progress in labor (LoPiL).

CS Indication	Dinoprostone (*n* = 36)	Misoprostol (*n* = 107)	Foley (*n* = 32)	All (*n* = 175)	*p*-Value
					0.0031 ^1^
NRFHRP	26 (72.2%)	98 (91.6%)	25 (78.1%)	149 (85.1%)	0.0363 ^1^
LoPiL	10 (27.8%)	9 (8.4%)	7 (21.9%)	26 (14.9%)	

^1^ Chi-square.

**Table 5:** Comparative analysis in three groups (Dinoprostone, Misoprostol, and Foley catheter: cesarean section indications within 48 h (nonreassuring fetal heart rate patterns (NRFHRP) and lack of progress in labor (LoPiL).

CS Indication	Dinoprostone (*n* = 102)	Misoprostol (*n* = 43)	Foley (*n* = 44)	All (*n* = 189)	*p*-Value
					0.0186 ^1^
NRFHRP	61 (59.8%)	24 (55.8%)	17 (38.6%)	102 (54.0%)	
LoPiL	41 (40.2%)	19 (44.2%)	27 (61.4%)	87 (46.0%)	

^1^ Chi-square.

## Data Availability

Data sharing is not applicable to this article due to the risk of the possibility of deanonymization.
